# 3D printed plates based on generative design biomechanically outperform manual digital fitting and conventional systems printed in photopolymers in bridging mandibular bone defects of critical size in dogs

**DOI:** 10.3389/fvets.2023.1165689

**Published:** 2023-03-30

**Authors:** Doris Baumgartner, Johannes Peter Schramel, Silvio Kau, Ewald Unger, Gunpreet Oberoi, Christian Peham, Matthias Eberspächer-Schweda

**Affiliations:** ^1^Movement Science Group, University Equine Hospital, Department for Small Animals and Horses, University of Veterinary Medicine Vienna, Vienna, Austria; ^2^Small Animals Surgery Department for Small Animals and Horses, University of Veterinary Medicine Vienna, Vienna, Austria; ^3^Department of Pathobiology, Institute of Morphology, University of Veterinary Medicine Vienna, Vienna, Austria; ^4^Center for Medical Physics and Biomedical Engineering, Medical University of Vienna, Vienna, Austria

**Keywords:** jaw, canine, critical size, osteosynthesis, additive manufacturing, customized endoprosthesis, biomechanical evaluation, autodesk fusion 360

## Abstract

Conventional plate osteosynthesis of critical-sized bone defects in canine mandibles can fail to restore former functionality and stability due to adaption limits. Three-dimensional (3D) printed patient-specific implants are becoming increasingly popular as these can be customized to avoid critical structures, achieve perfect alignment to individual bone contours, and may provide better stability. Using a 3D surface model for the mandible, four plate designs were created and evaluated for their properties to stabilize a defined 30 mm critical-size bone defect. Design-1 was manually designed, and further shape optimized using *Autodesk*^®^ *Fusion 360* (ADF360) and finite element analysis (FE) to generate Design-2. Design-4 was created with the *generative design* (GD) function from ADF360 using preplaced screw terminals and loading conditions as boundaries. A 12-hole reconstruction titanium locking plate (LP) (2.4/3.0 mm) was also tested, which was scanned, converted to a STL file and 3D printed (Design-3). Each design was 3D printed from a photopolymer resin (VPW) and a photopolymer resin in combination with a thermoplastic elastomer (VPWT) and loaded in cantilever bending using a customized servo-hydraulic mechanical testing system; *n* = 5 repetitions each. No material defects pre- or post-failure testing were found in the printed mandibles and screws. Plate fractures were most often observed in similar locations, depending on the design. Design-4 has 2.8–3.6 times ultimate strength compared to other plates, even though only 40% more volume was used. Maximum load capacities did not differ significantly from those of the other three designs. All plate types, except D3, were 35% stronger when made of VPW, compared to VPWT. VPWT D3 plates were only 6% stronger. *Generative design* is faster and easier to handle than optimizing manually designed plates using FE to create customized implants with maximum load-bearing capacity and minimum material requirements. Although guidelines for selecting appropriate outcomes and subsequent refinements to the optimized design are still needed, this may represent a straightforward approach to implementing additive manufacturing in individualized surgical care. The aim of this work is to analyze different design techniques, which can later be used for the development of implants made of biocompatible materials.

## Introduction

Critical size defects of the mandible often occur after fracture complications, osteonecrosis or after removal of mandibular tumors. Mandibular fractures in dogs represent up to 6% of all fractures (1–5). Most commonly mandibular fractures occur in the premolar and molar region (1) involving the first mandibular molar tooth (309/409) predominately (6). A previous study reported that most open fractures occurred in tooth-bearing regions and involved tooth roots (6) with mandibular first molar tooth being the most frequently involved also in pathological fractures (7). Osteonecrosis of the jaw is a well-known disease in human medicine, and a recent paper in veterinary medicine revealed a 40.9% involvement of the mandible in 14 cases. Its treatment often requires aggressive surgical removal of bone. Several benign and malignment tumors occur in the mandible (8) and the treatment may require segmental mandibular resection.

Although improvements in surgical techniques and perioperative care have enhanced the management of complex oral and maxillofacial fractures, adverse wound conditions, infection, or biomechanical instability can lead to major skeletal defects and malocclusion originally caused by high-energy trauma, tumor resection, teeth removal, revision surgery or other reasons (9, 10).

The reconstruction of such larger segmental defects in the mandibular bone, which can start as small as 15 mm (11), is a challenge in veterinary oral and maxillofacial surgery. This is especially true for the restoration of physiologic masticatory function and occlusion, as there is a high risk of malocclusion or non-union due to instability caused by mandibular drift, which is a commonly described complication after mandibulectomy and destabilization of the lower jaw (8, 12). The mandibular bone is a highly load-bearing bone of the face and acts as a long lever arm with bending forces as the primary acting force (13). During mastication, the bone is constantly subjected to tensile and compressive stresses. Tensile stress occur mostly on the alveolar margin, while compressive stress act mostly on the ventral border of the mandibles. The highest shear forces can be measured at the mandibular ramus, and the highest rotational forces at the symphysis. As a result of a mandibular fracture, the acting forces, together with contraction of the masticatory muscles, irrevocable lead to incomplete mouth alignment. The anatomical configuration and acting tensile and compressive surfaces of the bone must be considered in any reconstruction method. Fixation devices are strongest in tension and should therefore ideally be placed on the tension surface of the bone, causing all forces to act parallel to the long axis of the implant. In mandibular fractures, this location is along the alveolar margin and ventral border, where the basic biomechanical principle of tension band fixation applies (13). Since the principle is also based on the maximum compressive stresses at the ventral edge of the mandible and interfragmentary compression, it is difficult to apply to critical size defects (14).

The treatment of craniomaxillofacial defects presents many challenges due to the diverse tissue-specific requirements and complexity of the anatomical structures in this region involving bone, gingiva, teeth, nerves, and vascular structures (15). With the current reconstruction options in human medicine and the use of autologous bone grafts, oromandibular reconstruction can be highly successful (10) but still unsatisfactory (16–19). Medical modeling and 3D printing are already being used in three main strategies to restore both appearance and function to patients because of many opportunities they offer. For example, to produce three-dimensional models for pre-operative planning, prostheses, custom incision guides, fixation devices or scaffolds (10, 20).

Patient-specific implants (PSI) which are either CNC milled or 3D printed are currently used in multiple areas, but especially in human oral and maxillofacial surgery, i.e., total temporomandibular joint replacement, reconstruction of the maxillofacial skeleton, and orthognathic surgery (21). PSIs enable a more accurate reconstruction of maxillofacial defects, eliminating the usual complications seen in performed implants (22) and reducing surgical time (23).

3D printing is known to be an energy-efficient technology, both in terms of the manufacturing process and waste prevention. Some companies are already offering 3D printers on a lease basis, which allows clinics to take advantage of the latest advancements and reduce their costs in not having to purchase the 3D printing equipment (24) and can already be completed at an equal cost to conventional methods (25).

The complications and limitations seen in human medicine are even more serious in the field of oral surgery in small animals, where conventional plate systems may reach their limits. Due to the even smaller bone volume and the delicate structures in the mandibular bone (tooth roots, nerves, vessels), the fixation of normal metal plate/screw configurations may result in damage of these vital structures. The plates can sometimes not be perfectly matched to the bone with fixed screw holes in one row, and may damage the neurovascular system or tooth roots when the plate is inserted (14, 26). The stability of the bone-plate system may be severely compromised, as tooth trauma caused by the screws can lead to inflammation or infection, which in turn can cause fixation failure (27–29).

Customized plates can be built to minimize stress-risers and are manufactured in the exact shape of the bone, allowing for a potential better fit to the mandible and bone segment, as well as better bony integration and stability. Stress ranges can be minimized because the plate does not have to be bent beforehand to fit (30). Higher stress levels on the plate screws in the non-customized types can lead to faster stability loss and failure. Inadequate adaptation of the bone and plate can also lead to unintentional movement between the segments of the mandible and thereby impaired bone repair (31). There are only a few clinical studies and empirical data concerning the ideal appearance of 3D printed implants for optimal results. Scientific publications from Freitas et al. (32) and Bray et al. (33) used different designs but showed potential for improvement.

With this study, we aimed to establish a replicable workflow using *Autodesk*^®^ *Fusion 360* for the design of patient-specific bridging plate models as a potential fixation to treat a critical-size bone defect that occurs predominantly in the mandibular first molar region in dogs.

## Materials and methods

### Specimen preparation and CBCT scan

**Figure 1:**
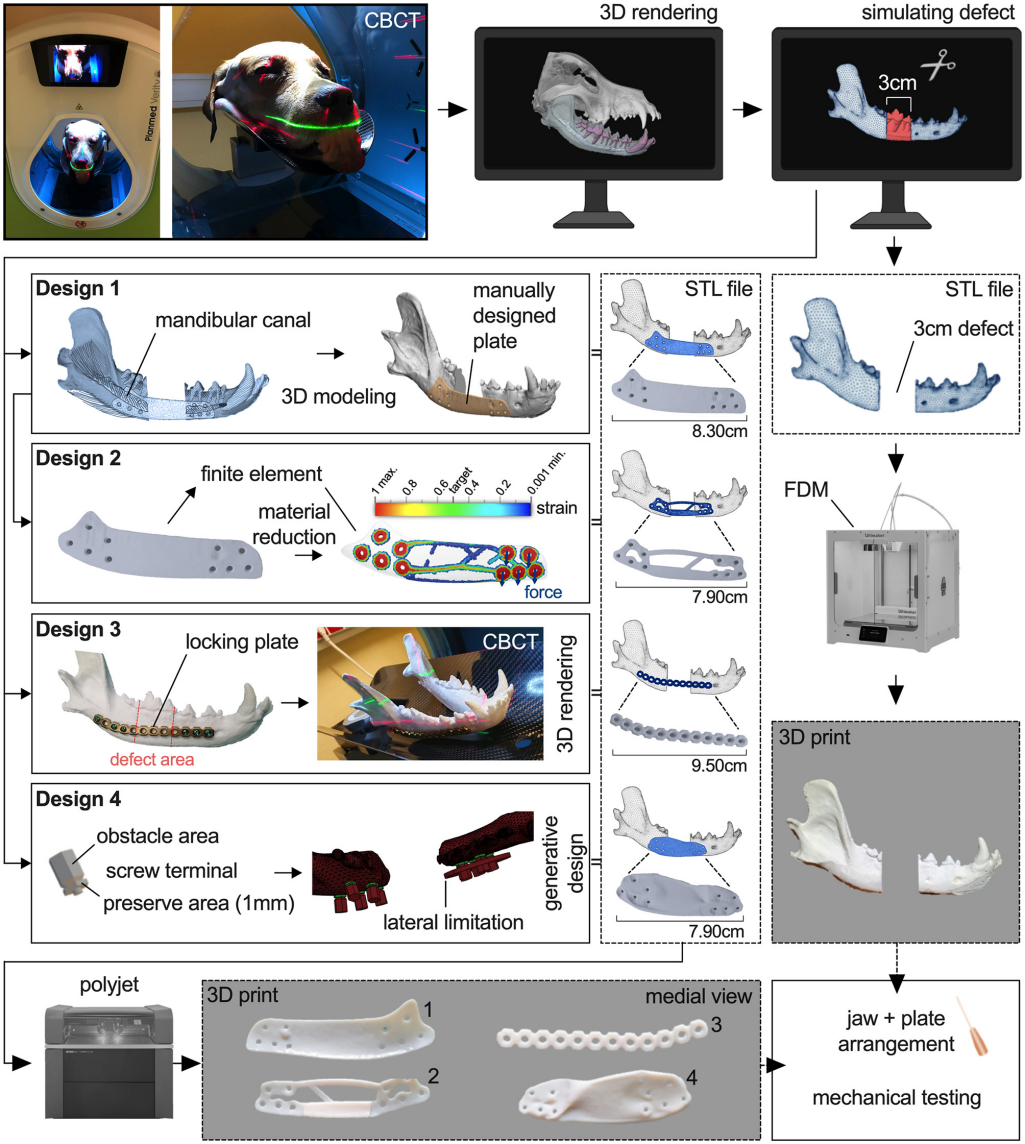
Workflow showing the process of printing a 3D model of a right canine mandible with a simulated 30mm defect and the development and printing of four different plate designs.

The head used as a model for *ex vivo* tests was derived from a freshly frozen adult dog carcass (Labrador, 3y, male, ~40 kg). The skull was placed and scanned in a dorsal position using cone-beam computed tomography (CBCT: *Planmed Verity*^®^*, Planmed OY, Helsinki, Finland*) acquiring sequential transverse images with a 0.2mm slice thickness, 96kV, 10mA. A 2ml syringe was placed between the upper and lower incisional region to reduce disruptive artifacts between teeth. Because of the head size, two scans (80 × 130 mm each) were necessary to obtain a complete model of the head. The stitched images were accepted when the result was labeled “good” (Software: *Planmeca Romexis*^®^*, Planmeca OY, Helsinki, Finland*). The images were reconstructed into a 3D digital surface model of the canine mandible using special software (*Amira, Thermo Fisher Scientific Inc., Waltham, Massachusetts, United States of America*). Tooth roots and the mandibular canal were highlighted to best possible avoid them during screw placement for future plate designs. A critical-sized defect of 30 mm rostro-caudal length was simulated with CAD software between the mesial root of the mandibular forth premolar tooth (408) and distal root of the mandibular first molar tooth (409) and excised in the right mandible. The generated cranial and caudal mandible parts were used for the design of the different plate models (Figure 1).

### Study design

In the study, 8 experimental groups with four different plate designs, each printed five times from two different polymers, were evaluated for their biomechanical properties.

### Plate design development

The obtained 3D model of the defect right mandible was used for the design of the first bridging plate (Design-1, D1) with certain general parameters, i.e., a thickness of 1 mm, like the smallest height of locking mini plates for mandibular reconstruction, a minimum of three 2 mm bicortical non-locking countersunk screws per side with at least two additional holes for auxiliary screw placement and at least one screw diameter distance from each other, and rounded edges to avoid soft tissue healing complications. Prohibited areas for screw placement were the alveolar margin, tooth roots and mandibular canal. If screw placement in the mandibular canal could not be avoided, screws were placed in the dorsal region whenever possible. Cranial and caudal boundaries were defined as the mental foramen and insertion of the masseter and digastricus muscle, respectively. The manual design process was carried out using the CAD software *Materialize 3-Matic 13.0 software (Materialize, Leuven, Belgium)*, which was made available as part of the collaboration with the Center for Medical Physics and Biomedical Engineering at the Medical University of Vienna. The 3D model of the mandible was imported into the software and cut in the rostral symphysis to leave the right mandible. An offset layer around the mandible model of 1 mm was created as base for the plate design to achieve a perfect alignment to the bone. Areas for possible screw placement were confirmed with CBCT scan and subjective assessment of spatial position of tooth roots, mandibular canal, and the ventral margin of the mandible. The outer contour of the plate was sketched on the offset layer. By extruding the subdivided sketch from the offset layer up to the model surface, all sections of the plate with a thickness of 1 mm were generated and joined together. The screw holes were then added and removed. The finished design was further smoothed to remove sharp edges and irregularities and reduce the triangle count of the STL file (Figure 1).

For the second design (Design-2, D2), the digital Design-1 plate was further investigated using finite element analysis in *ADF360* and manually optimized as deemed appropriate to reduce material using *3-Matic 13.0 software*. For simulation, two forces of 100N were applied to both distal screw holes in vertical direction parallel to the median plane of the jaw. Fixation constraint was attached to the two most proximal screw holes. The result shows regions with highest strain. A slider allows a selection of the percentage of material that can be removed or is needed. *ADF360* suggests a target mass of 30% of the original in the default setting to maximize the stiffness while reducing the mass of the part. For the optimization of D2, this recommendation was taken as a template to include the areas with higher strain in any case and remove those areas with no significant strain. In contrast, areas with higher forces measured were reinforced, including a bar at the inner ventral border between the cranial and caudal mandibles to improve compressive strength (Figure 1).

For better comparison, a Synthes^®^ 24-hole 2.4/3.0 mm reconstruction titanium locking plate cut into half was also placed on a 3D-printed bone model and shaped by one of the authors (ME-S), Dipl. AVDC, to fit the buccal surface of the mandible just dorsal to the mandibular canal and ventral to the tooth roots. The contoured Synthes^®^ locking plate (Design-3, D3) was scanned with CBCT. The plate was then 3D modeled using *Amira*. An appropriate threshold (2900 to 4884) was selected to mask the image voxels of the plate as best as possible and reduce artifacts of beam hardening. After selection, the model was refined to minimize the remaining artifacts while the surface was smoothed, using the original plate as a template (Figure 1).

**Figure 2:**
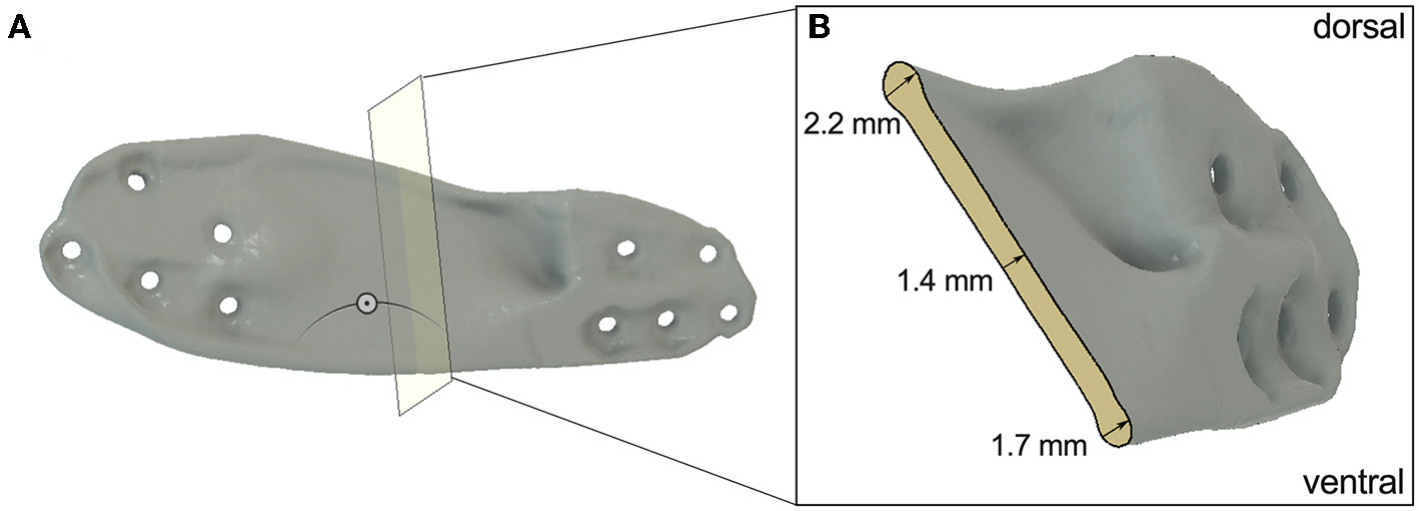
**(A)** Section analysis of D4 to show the thickness of the midsection of the plate. **(B)** Cross section of D4.

Design-4 (D4) was constructed using GD from *Autodesk*^®^ *Fusion 360*. An universal screw terminal was designed to provide the screw hole, preserved (mandatory) areas for the screw head and obstacle (keep out) areas for screwdriver access. The terminal has a 1 mm thick hexagonal body around the screw hole and an obstacle body with tapered bevel serving as input for the GD algorithm. Round shapes were avoided to reduce FE calculation effort. The terminals were placed at the same locations as the screw holes of D1 and D2. The mandibles were also defined as obstacle input for the GD. Two forces of 100N were applied to both distal screw holes in vertical direction parallel to the median plane of the jaw. Fixation constraint was attached to the two most proximal screw holes. By predefining obstacle and preserve regions and implementing loading forces, the software generated several designs from which to choose. After initial trials, another lateral boundary was added in the cranial region (Figure 1) to prevent extreme lateral bulging of the possible plate designs, but no general limit of 1 mm thickness was specified. No dorsal or ventral constraint was needed because the generated plate proposals did not go over the alveolar crest or ventral mandibular margin. A total of three designs were proposed, two of which also had protrusions medially into the defect, making them ineligible. The selected design was then refined by rounding or removing existing edges according to experience to avoid complications during wound healing. The thickness of the midsection of the finished design was between 1.4 and 2.2 mm (Figure 2).

### Additive manufacturing of the mandible and plate designs

The STL files of the cranial and caudal part of the virtual 3D model of the mandible were rapidly prototyped in-house by fused deposition modeling (FDM) using an Ultimaker S5 (Ultimaker, Utrecht, NL) 3D printer. The skeletal model was printed in Ultimaker ABS White with a layer resolution of 0.2 mm.

The plates were additively manufactured using a PolyJetTM Connex3 Objet500 printer (*Stratasys, EdenPrairie, MN, United States of America*) with a printing resolution of 16 microns and an accuracy of 30 microns. Each implant design was printed five times using two different photopolymers. This resulted in 20 plates printed in Vero Pure White RGD835 (VPW, *Stratasys Ltd., Eden Prairie, MN*), a rigid white material, and 20 plates made from a combination of VPW with TangoPlus FLX930 (VPWT, *Stratasys Ltd., Eden Prairie, MN*), a rubber-like material. There were two reasons for using these two different materials. On the one hand, we wanted to check the properties of the design regardless of the material. On the other hand, the moduli of elasticity of biomedical titanium alloys are much smaller than those of other metallic biomaterials (34). The elastic modulus measures the resistance of the material to elastic deformation. Low modulus materials stretch more when they are pulled (35). The mixture of TangoPlus FLX930 (TP) and VPW reduces the modulus of elasticity of the photopolymer and gives us the possibility to simulate the higher elasticity of titanium compared to a more rigid material. TP does not have an official modulus of elasticity, because of the difficulty to calculate it analytically (36), but mechanical parameters such as tensile strength and elongation at break can be used for comparison and are far less compared to VPW (37, 38).

Printing time of the plates was 4 h, and all the plates were printed in a single cycle. The plates were then cleaned manually to remove the gross support material (SUP706) and later flushed carefully using waterjet. The models were then placed in 2% sodium hydroxide solution for 30 min to dissolve the rest of the support material and rinsed with water.

### Plate fixation method

**Figure 3:**
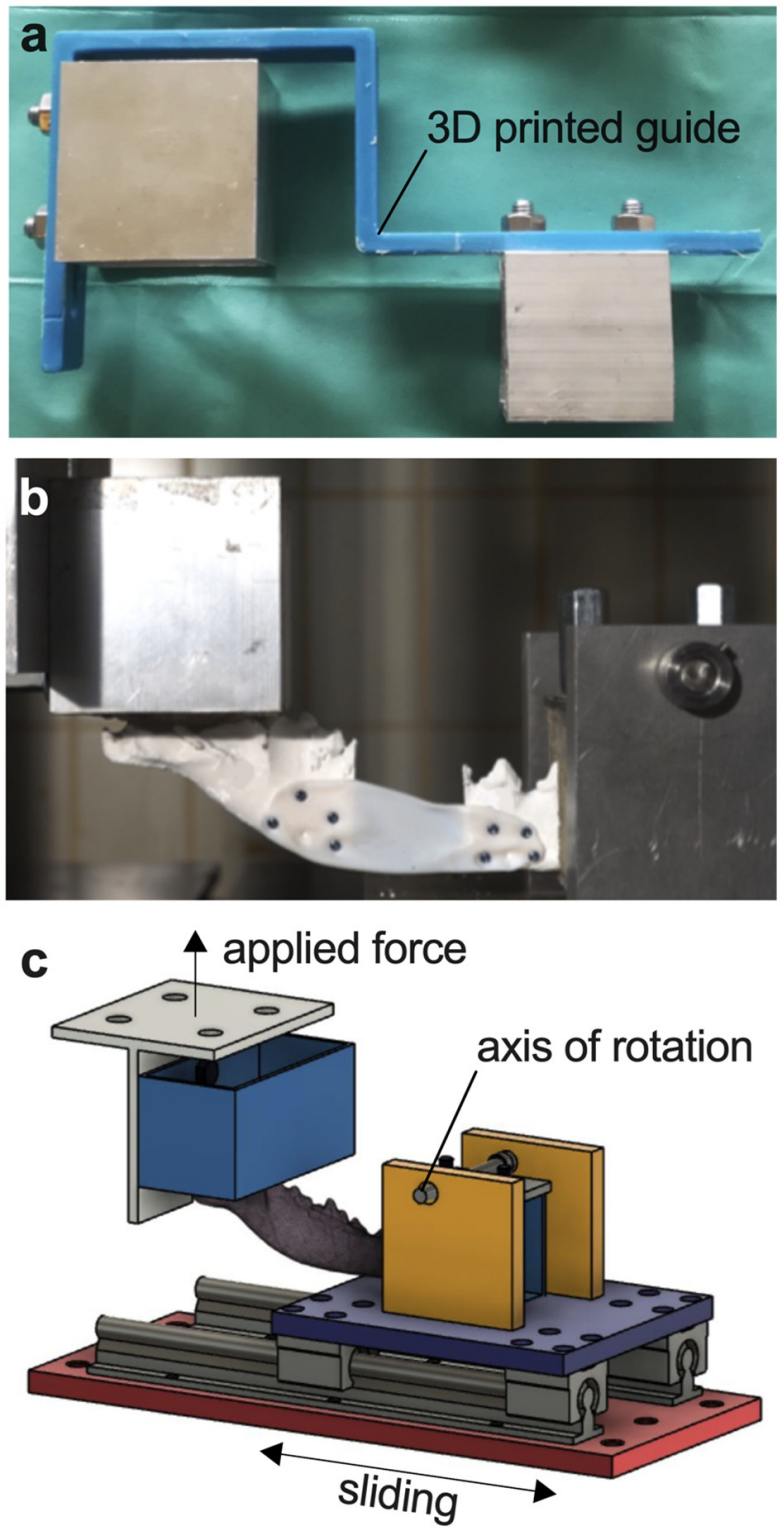
Servo-hydraulic testing system. **(A)** Custom 3D printed guide for correct positioning of the mandible. **(B)** Photograph of a fixated mandible-plate-construct in cantilever bending. **(C)** Illustration of cantilever bending of a mandible fixated at the ramus and canine teeth in the testing system with applied force perpendicular to the body of the mandible.

Before fixing the plates, the screw positions were marked on the printed models of the mandible parts using a Design-1 plate. Due to the adapted shape, positioning was only possible at one location, thus ensuring accurate marking. The correct distance between the cranial and caudal portions of the mandible was rechecked by subsequently measuring the 30 mm defect. The plates of Design-1, Design-2 and Design-4 were fixated with four screws per side using 2 mm-diameter bicortical non-locking screws. Design-3 was fixed with three conventional 2.4 mm titanium locking screws (threaded head, self-tapping, standard pitch) per side, but were not actually locked into the plate, because of missing locking threads. To ensure correct positioning of the rostral and caudal portions of the mandible for mechanical testing, a custom 3D printed guide was used (Figure 3A). The caudal end of the mandibular ramus, which contains the attachment sites for the masticatory muscles was fixed in rapid concrete with its coronoid process in the middle. Screws were used for further stabilization and rigid fixation in the mechanical testing system. The rostral portion with the canine in center was also fixed in rapid concrete to allow rotation of the rostral aspect during loading (Figure 3).

### Mechanical testing

3D printed mandibles and all plates were mounted in a custom-built test fixture modeled after a similar system used at the University of California at Davis William R. Pritchard Veterinary Medical Teaching Hospital and loaded in cantilever bending using a servo-hydraulic mechanical testing system, simulating physiological masticatory forces as closely as possible (Figures 3B, C). The load was applied perpendicular to the occlusal surface of the canines. All mandibular plate constructs were loaded in a single load-to-failure test under displacement control at 1 mm/s for 100 mm. Load and axial displacement were recorded. Each test was carried out to the end, even if maximum load had already been reached and the plate was bent or broken. Maximum load as point of failure and end of test was then evaluated using the data and video recordings.

### Data analysis

The data analysis was adapted from the study of Arzi et al. (14), comparing two conventional plating configurations for the same oral and maxillofacial problem (14). The yield for each mandibular construct was determined by detecting a deviation from linearity with a regression line. The stiffness of the plate-bone constructs before yielding was calculated as the slope of the middle third of the data between the start of the loading curve and the yielding of the construct. The failure of the construction was determined as the point of maximum loading. Stiffness after yielding was calculated as the slope of the middle third of the data between yielding and failure of the structure. Yield and failure loads and displacements were the respective values at the yield and failure points. The yield and failure energies were calculated as area under the load-deformation curve up to the yield point (YP) and up to the failure point. The mode of failure (plate failure or plate bending) was recorded immediately after the failure test. Photographs and video recordings were taken for each plate and examined after the test to verify the result.

### Statistical analysis

Statistical analyses and illustrations were performed using GraphPad Prism software version 9.0.0 (*GraphPad Software Inc., San Diego, CA, USA*). Descriptive statistics were calculated for all data and reported as mean ± standard deviation (SD). All data was tested for normal distribution using Shapiro-Wilk test. The non-parametric Mann–Whitney *U*-test was used for comparison with non-normally distributed data. Other data were analyzed using two-way ANOVA and Tukey's multiple comparisons with “load”, “stiffness”, and “displacement” as respective dependent variable and “plate design” as well as “plate material” as independent variables. All tests were performed with two-sided hypothesis tests and the significance level was set at *p* < 0.05.

## Results

### Plate fracture dominates mode of failure

**Figure 4:**
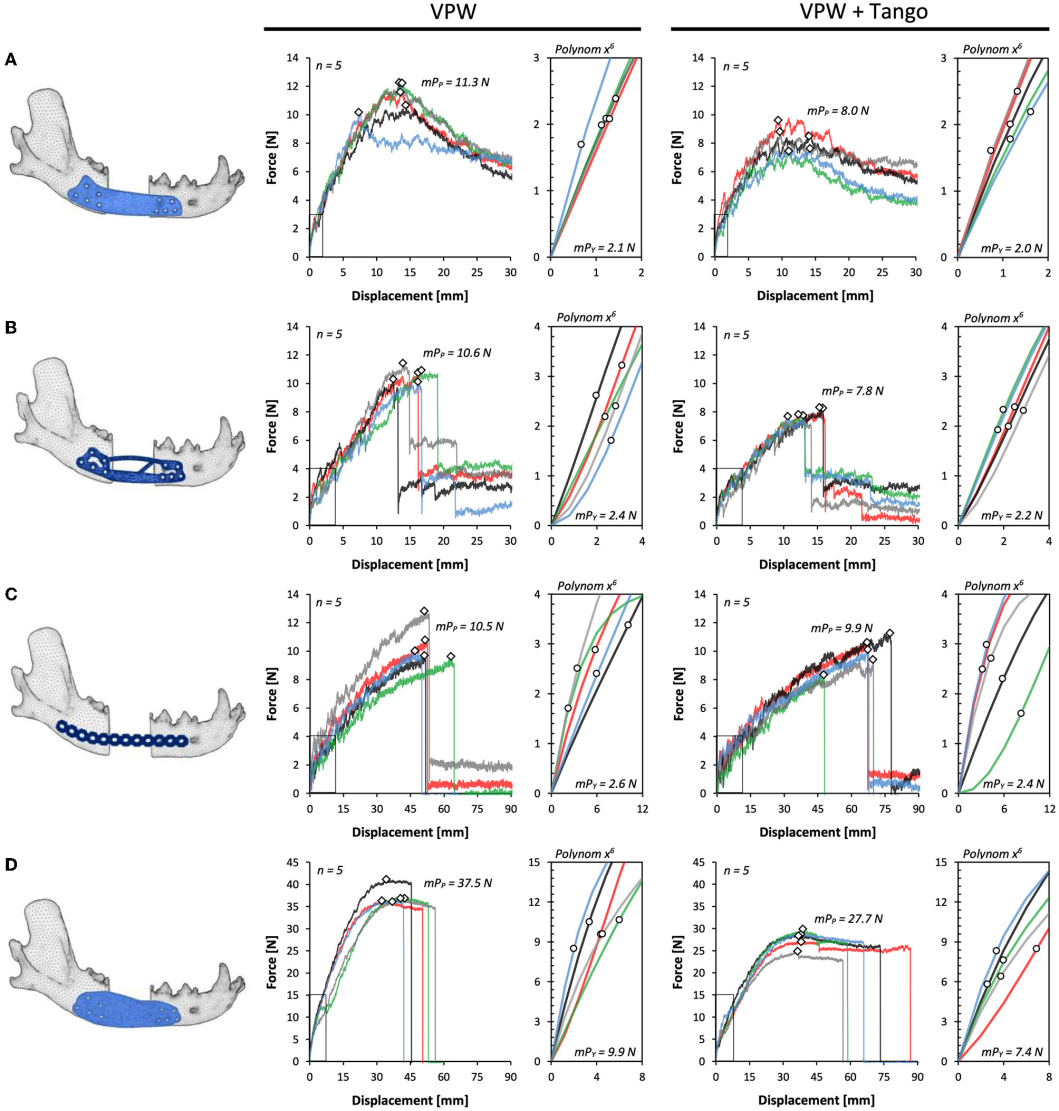
Force-displacement curves for plate designs D1–4 **(A–D)** comparing rigid (VPW) and combination of rigid + rubbery (VPW+Tango) 3D printing materials. Yield points (circles) are shown in respective axis cutouts of start point linear variable interrelation; peak force/failure points are displayed as rhomb. mP_p_, mean peak force point; mP_y_, mean point of yield.

**Figure 5:**
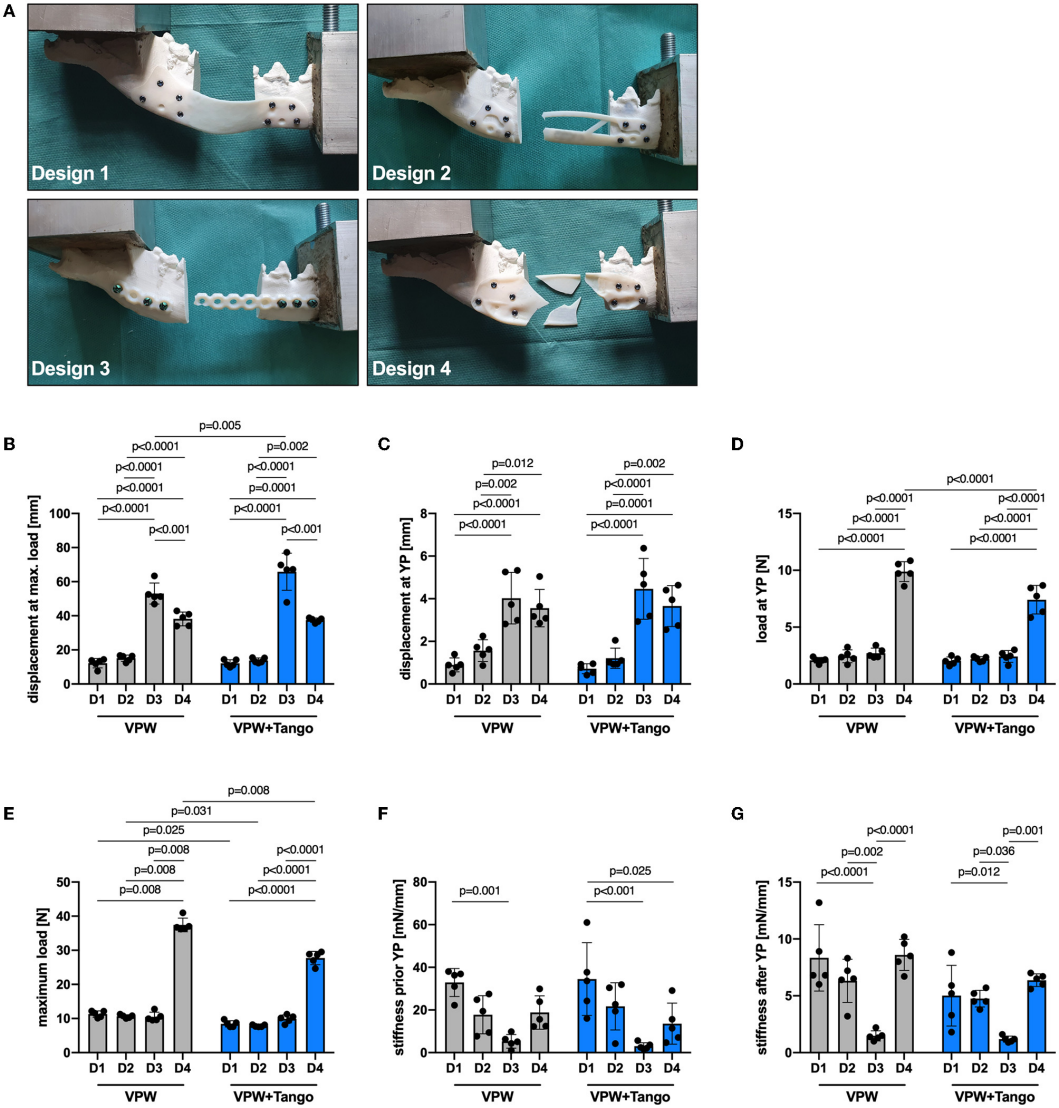
Mechanical properties of plate designs D1–4 comparing rigid (VPW) and combination of rigid + rubbery (VPW+Tango) 3D printing materials. **(A)** Failure modes representative for different plate designs. Note bending deformation in D1 being different to breakage in other designs. **(B)** Displacement at maximum load, **(C)** displacement at yield point (YP), and **(D)** force load at YP; ordinary two-way ANOVA, Tukey's multiple comparisons. **(E)** Maximum force load at point of failure; two-tailed Mann-Whitney-U test (VPW D1–3 vs 4, VPW D4 vs VPW+T D4) and ordinary two-way ANOVA with Tukey's multiple comparisons for all other variable combinations. **(F, G)** Material stiffness prior and after YP; ordinary two-way ANOVA, Tukey's multiple comparisons. **(B–G)** *n* = 5 replicates/design and material; bars represent mean, error bars represent ± SD.

There was no breakage under load of any printed mandibular parts or screws in any design construct. In all D1 VPW and VPWT tests, no fracture but deformation of the plate happened at maximum force (Figure 4A). Four D1 VPW plates finally failed at the end of the mechanical testing (Supplementary Video 1), whereas one D1 VPW and all VPWT plates just bent into the defect area with no apparent fracture (Supplementary Video 2). All D2 VPW and VPWT plates failed fracturing along the screw holes closest to the caudal bone margin of the defect (Supplementary Video 3). D3 constructs failed with fracture of the plate in the caudal section between the 4th and 5th screw hole (Supplementary Video 4). Constructs with D4 all failed with multiple fragmentation of the middle part of the plate without involvement of the screw holes (Supplementary Video 5, Figure 5A).

### Mechanical resilience is down to plate design and plate material

All mandibular models were 3D printed with an average length of 175 mm together with a 30 mm rostro-caudal defect between the mesial root of the mandibular forth premolar tooth and distal root of the first molar tooth. Therefore, and due to the standardized jaw and plate fixation with a guided approach, the length of the moment arm was the same in all groups. The displacement at yielding and failure was highest in D3 for both VPW and VPWT constructs (Figure 4C), followed by D4 (Figure 4D) without significant difference. D2 and D1 showed the smallest displacement (Figures 4A, B) compared to D3 and D4 at maximum load (Figure 5B) and YP (Figure 5C). Load at YP was highest in all D4 constructs in any the material, with a significant difference to D1, 2 and 3. The load required to reach the YP was significantly higher for all D4 VPW plates compared to D4 VPWT plates (Figure 5D).

The measured values of maximum load, which determine the point of failure, showed little variance within the individual test group. Except for D3, VPW plates withstood more than 30% force up to the point of failure compared to VPWT plates. Design-4 was more than three times stronger than D1, D2 and D3 in both materials. The maximum load between D1, D2 and D3 was not significantly different for either material (Figure 5E).

The D1 plates were significantly stiffer before yielding compared to D4 made of VPWT, and D3 in any material which showed the lowest stiffness. The distribution of the individual values regarding the stiffness just before the yield point was very high especially in D1 plates made of VPWT material (Figure 5F). After the yield point, the stiffness of the D3 plates was significantly lowest in both materials, compared to the other three designs, and showed the least distribution like before yielding (Figure 5G, Supplementary Table 1).

The D4 constructions made of VPW needed about four times the energy to yield compared to D1 (4.7), D2 (4.1) and D3 (3.7). The constructs made of VPWT D4 also required more than three times the energy to yield, compared to D1 (3.7), D2 (3.4) and D3 (3.1). Energy to failure was highest for the D4 designs in both materials: 2.7 (VPW) and 1.7 (VPWT) times more than D3, 11 (VPW) and 10.2 (VPWT) times more than D1 and 10.4 (VPW a. VPWT) times more than D2 (Supplementary Table 2).

## Discussion

Additive manufacturing in medicine has the advantage of finding a solution to a specific problem in a completely individual way that can fit complex anatomy and mimic bone morphology. The bridging of large defects in canine mandibles can be a surgical problem due to the structural conditions and the resulting distribution of forces. The use of titanium locking reconstruction plates and locking miniplates was already tested in a study by Arzi et al. (14). A single locking plate (LP) and a combination of LP and miniplate were tested biomechanically in comparison to an intact mandible. Neither of the fixations was anywhere near as strong as the intact bone. Although reconstruction with two plates was more stable and stiffer, tooth roots and the mandibular canal were injured significantly more often. Because this study simulated a worst-case scenario, they concluded that both methods are likely to provide adequate stability but still affect vital structures and are therefore limited in their application. With additive manufacturing, it is possible to customize screw placement in order to avoid damaging vital structures and configurate plate sizes for different breeds and weight classes (32). In addition, both the placement and the number of screws placed in the mandibular canal area negatively affects the biomechanical variables of the reconstructed mandible (39). To date, there are no exact specifications for the plates, which makes it difficult to take the first steps toward their development. In this study, we wanted to investigate the benefits of using generative design software to develop such a suitable plate design compared to a manually created design.

When developing the first plate design, the main consideration was the anatomical conditions and the areas to be avoided. In addition, the plate should be positioned near the tensile zone. The thickness of the plate was 1 mm, like the smallest miniplate system available, to minimize the material required and obtain a plate that conforms to the bone as well as possible. As demonstrated in humans lateral oromandibular reconstruction, plate geometry, including profile height and plate contour, plays an important role in the successful surgical site healing without external plate exposure (40). The number of screws was set at a minimum of three per side. The placement and final number of screw holes was determined based on the complex anatomical conditions and limitations avoiding critical structures, preventing possible screw-induced dental trauma and reduce risk of infection and inflammation (27–29). They were placed with an appropriate distance between the defect and the edge of the plate, but this was determined from surgical experience only. The screw holes were designed offset from each other to assist with stress distribution. The biomechanical testing of the plate-bone-constructs was performed only by bending, since dogs are hardly able to perform lateral or forward and backward movements (41). The invented parameters for the first design resulted in a plate with perfect screw placement but low maximum force capacity. The stiffness of Design-1 before the yield point was the highest compared to the other designs, but with the smallest yield force and yield displacement. This caused a rapid elastic deformation of the plates, which was visible by bending medially into the defect area. Although the maximum force to the point of failure is comparable to D2 and D3, the deformation of the plate happened faster. This can be clearly seen in the tests of the more elastic material mixture VPWT.

The verification and optimization of the first design using FE showed that exact force distribution within the plate cannot be determined in advance by own considerations. In some edge areas as well as in areas within the slab, no large force loads were measured, so that material could be dispensed with. In addition, the material was reinforced at points where more stress within the design was detected. The dorsal arch was thickened, and a flat bar was added medially at the ventral edge between the rostral and caudal ends of the defect to resist the measured tension and compression forces that occur when a force is applied perpendicular to the longitudinal axis. Due to the reinforcements within the plate, the material reduction achieved was basically negligible at < 2%. As intended, the change in design did not significantly affect the maximum force at the point of failure. However, during the biomechanical tests, it was found that a design flaw during optimization in the rear plate area resulted in a predetermined breaking point. When a plate is bent, increased mechanical stresses occur especially at the edge (42), which led to breakage at the corner in all plates of the second design in both materials, with or without the inclusion of the first caudal screw hole (Figure 5A). It is reasonable to assume that D2 would have withstood more force if this flaw had not been inadvertently inserted into the second design, since it is not the deformation of the plate as in D1, but the fracture at this point that limits the maximum force that the structure can withstand. D3, the reconstruction LP, was significantly more dimensionally stable than D1 and D2 despite a similar maximum force and showed a similar large displacement at yield point as D4. Unlike all other designs, the maximum force of D3 did not differ between VPW and VPWT. The only difference was in the displacement to the point of failure. This showed, that although we were able to prevent screws from injuring vital structures with our manually designed plates, we were unable to achieve any real improvement in the load-bearing capacity and mechanics of the reconstructed mandibles compared to regularly used LPs. In comparison, however, about 20% less material was used for D1 and D2.

In addition, the stiffness of D1 had a high variance in the results, which may compromise the interpretation. Especially whether the observed differences are statistically significant. It may also be more difficult to make accurate predictions or generalize results to a larger test group. Stiffness was determined by calculations depending on the individual data of each test. Thus, the uniformity of the shape of the force-displacement curves influences the variance of the values. The stiffness of the plate-bone constructs before yielding was calculated as the slope of the middle third of the data between the start of the loading curve and the yielding of the construct. Stiffness after yielding was calculated as the slope of the middle third of the data between yielding and failure of the structure. Each plate in D1 began to flex either inward or outward after a short time. The yield point and maximum load were reached most quickly in the D1 tests and after the least amount of displacement. Therefore, the number of single values used to calculate the stiffness was lower than for the other designs. This resulted in a wide distribution of the calculated values.

Screws for D4 were placed at the same locations as in D1 and D2 for better comparison between the software design and the manual design. Due to the property of the photopolymers used and the small thickness of the plates (D1, D2), we have omitted the design of the threads inside the plates, unlikely to titanium locking plates. Normally, plate fixation with locking screws is more resistant to screw failure (43–47). Nevertheless, we were able to show that no construct yielded because of screw failure but due to fracture of the plate. The reason for this is probably that the screws were made of metal and not also of photopolymer. Although compared to all others, mandible-plate constructs with D4 were over three times stronger without screw failure, it is reasonable to assume that the mode of failure will be different for printed titanium plates, especially in the screw-plate area. For this reason, a thread should possibly be integrated for future designs to ensure better stability, which should be done during finishing process to achieve highest accuracy.

A study conducted from Freitas et al. (32) also dealt with the bridging of larger bone defects in the mandible and a solution by rapid prototyping of special plates with locking screws (32). However, a general plate was designed for two different weight classes and not individually adapted to the bone. In addition, the defects were all ≤ 10 mm. Compared to normal bite forces, the plate-bone systems showed a 10 to over 40-fold resistance at low, and a 2 to 9-fold resistance at very high chewing activity. Thus, we can assume that a customized 3D printed plate, already FE-tested and specially designed by generative design in advance, will possibly lead to similar stability with further material reduction.

Since the software works with predefined limits and rules, these must be described as accurate as possible. During the initial design development, it was apparent that a lateral limit also needed to be specified to prevent excessive excursions of the plate. In addition, it was necessary to refine the final design by rounding the edges and removing any ridges which could potentially lead to healing complications and plate extrusion (40). The screw terminals were well integrated into the plate design, although the hexagonal surface shape that the software starts the design with could be improved. A STL file is a triangular representation of a three-dimensional surface geometry. The higher the triangle count of the mesh, the more accurate the model could be. Flat surfaces need less triangles as approximation and lead to faster FE calculation. In this study, the size and resolution of the screw holes were limited by the 3D printing process, and the hexagonal shape was sufficient. However, if more precise round structures, such as threads, are required, this must be done subsequently by cutting out the hole.

The introduction of additive manufacturing in a clinical setting, especially for craniomaxillofacial implants, requires tools that are sufficiently precise and accurate to match patient-specific and anatomical free-form geometries (48). CBCT is the latest technology in veterinary diagnostic imaging, having been used in human medicine as state of the art in dentistry and oral and maxillofacial surgery for several years. Compared to conventional CT, CBCT provides significantly more detailed high-resolution 3D images, especially in hard tissue such as bone and teeth. The sectional images taken can be segmented (multiplanar reformation), saved, and exported as STL files for further orthodontic or prosthetic planning and 3D printing. This reduces time between imaging, designing together with planning and finally printing of the implants.

Guidelines are needed for selecting viable results and potential refinements of the optimized design. Special additions for bone grafting, which are often needed in large segmental bone defects (46) could also be easily incorporated into the design subsequently to improve functionality and bone healing.

## Study limitations

Only a limited statement can be made about the properties of 3D printed titanium plates in these constructions, as the tests were carried out with plates made of photopolymers. Nevertheless, it was found that the designs behaved similarly regardless of the material, so some predictions can be made about the properties of the plates made of titanium. Another limitation of the study is that the series of experiments was only performed on the mandible of a single dog, which was the size of a Labrador, making it easier to avoid tooth roots than in mandibles of smaller dogs. However, since the software and generative design program can be fully customized and the design depends only on the placement of the screws, it can be transferred to other individuals and thus weight classes.

## Conclusion

*Autodesk*^®^ *Fusion 360 generative design* is feasible for customized plate design providing maximum load capacity with reduced material effort. The proposed method eases plate construction by generating multiple solutions suitable for additive manufacturing. Compared to a manual plate design, it requires less time and can withstand higher maximum load and displacement than conventional (LP) or manually designed plates. Further tests with plates made of titanium fixed to bone must be carried out to be able to make an accurate statement about the force capacity and fracture behavior of *generatively designed* plates.

## Data availability statement

The original contributions presented in the study are included in the article/Supplementary material, further inquiries can be directed to the corresponding author.

## Ethics statement

Ethical review and approval were not required for the study because the used specimen was obtained from a cadaver. Cause of death was unrelated to this study. Written informed a general written consent for educational and scientific use of carcasses was existing.

## Author contributions

ME-S and JS conceived of the presented idea and designed the study with the help of DB, CP, and EU. SK did the cadaver sampling and analyzed the data and conducted statistical tests. DB and ME-S did the CBCT scans. DB and JS performed all computations to obtain 3D jaw elements and different plate designs. DB with the help of JS, EU, and GO did the 3D printing. Mechanical testing was performed by DB, JS, and CP. SK prepared all figures and tables with input from DB. ME-S, JS, and CP supervised the work, contributed to the interpretation of the results, and worked on the manuscript. DB wrote the main manuscript with the support of SK and ME-S. All authors contributed to the article and approved the submitted version.

## References

[1] Umphlet RC Johnson AL. Mandibular fractures in the dog. A retrospective study of 157 cases. Vet Surg. (1990) 19:272–5. 10.1111/j.1532-950X.1990.tb01184.x 2382396

[2] Wong W. A survey of fractures in the dog and cat in Malaysia. Vet Record. (1984) 115:273–4. 10.1136/vr.115.11.273 6495579

[3] Phillips IR. A survey of bone fractures in the dog and cat. J Small Anim Practice. (1979) 20:661–74. 10.1111/j.1748-5827.1979.tb06679.x 547112

[4] Kitshoff AM Rooster H de Ferreira SM Steenkamp G. A retrospective study of 109 dogs with mandibular fractures. Vet Comp Orthopaedics Traumatol. (2013) 26:1–5. 10.3415/VCOT-12-01-0003e 23111902

[5] Smith MM Kern DA. Skull trauma and mandibular fractures. Vet Clin Small Anim Prac. (1995) 25:1127–48. 10.1016/S0195-5616(95)50108-1 8578630

[6] Scherer E Hetzel S Snyder CJ. Assessment of the role of the mandibular first molar tooth in mandibular fracture patterns of 29 dogs. J Vet Dent. (2019) 36:32–9. 10.1177/0898756419846183 31138050 PMC6953385

[7] British Small Animal Veterinary Association. BSAVA Manual of Canine and Feline Dentistry. Quedgeley, Gloucs: British Small Animal Veterinary Association (2007).

[8] Verstraete FJ. Mandibulectomy and maxillectomy. Vet Clin Small Anim Prac. (2005) 35:1009–39. 10.1016/j.cvsm.2005.03.005 15979523

[9] Reichert JC Saifzadeh S Wullschleger ME Epari DR Schütz MA Duda GN. The challenge of establishing preclinical models for segmental bone defect research. Biomaterials. (2009) 30:2149–63. 10.1016/j.biomaterials.2008.12.050 19211141

[10] Kumar BP Venkatesh V Kumar KA Yadav BY Mohan SR. Mandibular Reconstruction: Overview. J Maxillofac Oral Surg. (2016) 15:425–41. 10.1007/s12663-015-0766-5 27833334 PMC5083680

[11] Huh J-Y Choi B-H Kim B-Y Lee S-H Zhu S-J Jung J-H. Critical size defect in the canine mandible. Oral Surg Oral Med Oral Pathol Oral Radiol Endod. (2005) 100:296–301. 10.1016/j.tripleo.2004.12.015 16122656

[12] Bar-Am Y Verstraete FJ. Elastic training for the prevention of mandibular drift following mandibulectomy in dogs: 18 cases (2005-2008). Vet Surg. (2010) 39:574–80. 10.1111/j.1532-950X.2010.00703.x 20459496

[13] Tobias KM Johnston SA. Veterinary Surgery: Small Animal. Missouri: Saunders (2012).

[14] Arzi B Stover SM Garcia TC Leale DM Verstraete FJ. Biomechanical evaluation of two plating configurations for critical-sized defects of the mandible in dogs. Am J Vet Res. (2016) 77:445–51. 10.2460/ajvr.77.5.445 27111011

[15] Elsalanty ME Zakhary I Akeel S Benson B Mulone T Triplett GR. Reconstruction of canine mandibular bone defects using a bone transport reconstruction plate. Ann Plast Surg. (2009) 63:441–8. 10.1097/SAP.0b013e31818d130c 19770704 PMC2811127

[16] Komisar A. The functional result of mandibular reconstruction. Laryngoscope. (1990) 100:364–74. 10.1288/00005537-199004000-00007 2181219

[17] Kuriloff DB Sullivan MJ. Mandibular reconstruction using vascularized bone grafts. Otolaryngol Clin North Am. (1991) 24:1391–418. 10.1016/S0030-6665(20)31043-4 1792077

[18] Jønsson B Siemssen SJ. Arced segmental mandibular regeneration by distraction osteogenesis. Plast Reconstr Surg. (1998) 101:1925–30. 10.1097/00006534-199806000-00023 9623838

[19] Ayoub AF Richardson W Koppel D Thompson H Lucas M Schwarz T. Segmental mandibular reconstruction by microincremental automatic distraction osteogenesis: an animal study. Br J Oral Maxillofacial Surg. (2001) 39:658. 10.1054/bjom.2001.0658 11601816

[20] Nyberg EL Farris AL Hung BP Dias M Garcia JR Dorafshar AH. 3D-printing technologies for craniofacial rehabilitation, reconstruction, and regeneration. Ann Biomed Eng. (2017) 45:45–57. 10.1007/s10439-016-1668-5 27295184 PMC5154778

[21] Huang MF Alfi D Alfi J Huang AT. The use of patient-specific implants in oral and maxillofacial surgery. Oral Maxillofac Surg Clin North Am. (2019) 31:593–600. 10.1016/j.coms.2019.07.010 31481289

[22] Alasseri N Alasraj A. Patient-specific implants for maxillofacial defects: challenges and solutions. Maxillofac Plast Reconstr Surg. (2020) 42:15. 10.1186/s40902-020-00262-7 32467823 PMC7239988

[23] Tran KL Mong ML Durham JS Prisman E. Benefits of patient-specific reconstruction plates in mandibular reconstruction surgical simulation and resident education. J Clin Med. (2022) 11:5306. 10.3390/jcm11185306 36142953 PMC9501640

[24] Choonara YE Du Toit LC Kumar P Kondiah PP Pillay V. 3D-printing and the effect on medical costs: a new era? Expert Rev Pharmacoecon Outcomes Res. (2016) 16:23–32. 10.1586/14737167.2016.1138860 26817398

[25] Truscott A Zamani R Akrami M. Comparing the use of conventional and three-dimensional printing (3DP) in mandibular reconstruction. Biomed Eng Online. (2022) 21:18. 10.1186/s12938-022-00989-6 35305669 PMC8934485

[26] Greiner CL Verstraete FJ Stover SM Garcia TC Leale D Arzi B. Biomechanical evaluation of two plating configurations for fixation of a simple transverse caudal mandibular fracture model in cats. Am J Vet Res. (2017) 78:702–11. 10.2460/ajvr.78.6.702 28541156

[27] Brisceno CE Rossouw PE Carrillo R Spears R Buschang PH. Healing of the roots and surrounding structures after intentional damage with miniscrew implants. Am J Ortho Dentofacial Orthop. (2009) 135:292–301. 10.1016/j.ajodo.2008.06.023 19268826

[28] Boudrieau RJ. Maxillofacial Fracture Repair Using Miniplates and Screws. Amsterdam: Elsevier (2012), 293–308.

[29] Marretta SM. Maxillofacial Fracture Complications. Amsterdam: Elsevier (2012), 333–41.

[30] Gutwald R Jaeger R Lambers FM. Customized mandibular reconstruction plates improve mechanical performance in a mandibular reconstruction model. Comput Methods Biomech Biomed Engin. (2017) 20:426–35. 10.1080/10255842.2016.1240788 27887036 PMC5359746

[31] Ramos VF Pinto LA Basting RT. Force and deformation stresses in customized and non-customized plates during simulation of advancement genioplasty. J Craniomaxillofac Surg. (2017) 45:1820–7. 10.1016/j.jcms.2017.08.021 28935483

[32] Freitas EP de Rahal SC Shimano AC da Silva JV Noritomi PY El-Warrak AO. Bridging plate development for treatment of segmental bone defects of the canine mandible: mechanical tests and finite element method. J Vet Dent. (2016) 33:18–25. 10.1177/0898756416639191 27487652

[33] Bray JP Kersley A Downing W Crosse KR Worth AJ House AK. Clinical outcomes of patient-specific porous titanium endoprostheses in dogs with tumors of the mandible, radius, or tibia: 12 cases (2013-2016). J Am Vet Med Assoc. (2017) 251:566–79. 10.2460/javma.251.5.566 28828951

[34] Niinomi M. Mechanical properties of biomedical titanium alloys. Mat Sci Eng A. (1998) 243:231–6. 10.1016/S0921-5093(97)00806-X36061649

[35] Jones DR Ashby MF. Elastic Moduli. In:Ashby MF Jones DR, editors. Engineering Materials 1: An Introduction to Properties, Applications and Design. Amsterdam: Butterworth-Heinemann (2019). p. 31–47.

[36] Abtan AA Richardson RC Thomas B. Analyzing the 3D printed material Tango plus FLX930 for using in self-folding structure. In: ICSAE 2016 Conference Program 2016 International Conference for Students on Applied Engineering 20-21 October 2016, Newcastle Upon Tyne: IEEE. p. 114–8.

[37] Stratasys Ltd,. Vero Family - EN Data Sheet PolyJet Material. Eden Prairie, MN. (2021). Available online at: https://stratasysstorage01.file.core.windows.net/ssys-websites-files-prod/Public1/Materials/Polyjet/Vero%20Family%20(Rigid)/Vero%20Family%20-%20EN%20Data%20Sheet%20PolyJet%20Material.pdf?sv=2017-04-17&sr=f&sig=ecF8TFPzU4Y2yWuWtSgYE8ceaNfEL7nD4bHUajXMBW8%3D&st=2022-12-24T13%3A34%3A38Z&se=2023-12-25T13%3A34%3A38Z&sp=rwl (accessed December 25, 2022).

[38] Stratasys Ltd,. Tango - EN Data Sheet. Eden Prairie, MN. (2018). Available online at: https://stratasysstorage01.file.core.windows.net/ssys-websites-files-prod/Public1/Materials/Polyjet/Tango%20(Rubberlike)/Tango%20-%20EN%20Data%20Sheet.pdf?sv=2017-04-17&sr=f&sig=zsWs%2BSQ6jqfUNxiqR2JeCmRVxHNUTS%2F13uIUZvzR8us%3D&st=2022-12-24T13%3A39%3A02Z&se=2023-12-25T13%3A39%3A02Z&sp=rwl (accessed December 25, 2022).

[39] Kot CC Verstraete FJ Garcia TC Stover SM Arzi B. Biomechanical evaluation of locking versus nonlocking 2.0-mm malleable L-miniplate fixation of simulated caudal mandibular fractures in cats. Am J Vet Res. (2022) 83:43. 10.2460/ajvr.22.03.0043 35895785

[40] Blackwell KE Lacombe V. The bridging lateral mandibular reconstruction plate revisited. Arch Otolaryngol Head Neck Surg. (1999) 125:988. 10.1001/archotol.125.9.988 10488984

[41] Harvey CE Emily PP. Small Animal Dentistry. St Louis, MO: Mosby. (1993).

[42] Lackmann J. Stresses in bars and beams. In:Grote K-H Feldhusen J, editors. Dubbel: Taschenbuch für den Maschinenbau. Berlin, Heidelberg: Springer Berlin Heidelberg (2007, C7–30.

[43] Mehta RP Deschler DG. Mandibular reconstruction in 2004: an analysis of different techniques. Curr Opin Otolaryngol Head Neck Surg. (2004) 12:288–93. 10.1097/01.moo.0000131444.50445.9d 15252248

[44] Boudrieau RJ Mitchell SL Seeherman H. Mandibular reconstruction of a partial hemimandibulectomy in a dog with severe malocclusion. Vet Surg. (2004) 33:119–30. 10.1111/j.1532-950X.2004.04019.x 15027973

[45] Klotch DW Gal TJ Gal RL. Assessment of plate use for mandibular reconstruction: has changing technology made a difference? Otolaryngol Head Neck Surg. (1999) 121:388–92. 10.1016/S0194-5998(99)70226-3 10504593

[46] Wong R Tideman H Kin L Merkx M. Biomechanics of mandibular reconstruction: a review. Int J Oral Maxillofac Surg. (2010) 39:313–9. 10.1016/j.ijom.2009.11.003 19944568

[47] Alpert B Gutwald R. Schmelzeisen R. New innovations in craniomaxillofacial fixation: the 20 lock system. Keio J Med. (2003) 52:120–7. 10.2302/kjm.52.120 12862364

[48] Akmal JS Salmi M Hemming B Teir L Suomalainen A Kortesniemi M. Cumulative inaccuracies in implementation of additive manufacturing through medical imaging, 3D thresholding, and 3D modeling: a case study for an end-use implant. Appl Sci. (2020) 10:2968. 10.3390/app10082968

