# Supplementary material for: 3D printed plates based on generative design biomechanically outperform manual digital fitting and conventional systems printed in photopolymers in bridging mandibular bone defects of critical size in dogs
Source: Front Vet Sci. 2023 Mar 30;10:1165689. doi: 10.3389/fvets.2023.1165689 (PMC10098091; doi:10.3389/fvets.2023.1165689)
Supplement: Supplementary file 2 [file Table_2.PDF]

Supplementary Table 2 – Median (range) values for mechanical test variables from a single-load-to-failure test of 3D printed mandible models with an experimentally created critical sized defect, reconstructed with four different plate designs. Each plate design was tested five times in two different materials (VPW, VPWT).

	**Group VPW**			
**Variable**	**Design 1 (n=5)**	**Design 2 (n=5)**	**Design 3 (n=5)**	**Design 4 (n=5)**
**Prior to yield**				
**Stiffness (mN/mm)**	32.9 (22.2-38)	17.8 (7.7-27.5)	5.3 (1-9.8)	18.8 (12.2-29.9)
**Yield**				
**Displacement (mm)**	0.9 (0.5-1.4)	1.6 (0.9-2.3)	4 (3-5.3)	3.6 (2.9-5)
**Load (N)**	2.1 (1.7-2.4)	2.4 (1.7-3.2)	2.7 (2.3-3.4)	9.9 (8.6-10.9)
**Energy (N*mm)**	1.5 (0.6-2.7)	3.2 (1.6-4.1)	8.4 (5.9-10.4)	22.9 (15.8-34.2)
**After Yield**				
**Stiffness (mN/mm)**	8.3 (6-13.2)	6.3 (3.2-8.3)	1.5 (1-2.2)	8.6 (6.7-10.2)
**Failure**				
**Displacement (mm)**	12.2 (7.5-14.4)	15.1 (12.4-16.7)	53 (46.9-63.4)	38.2 (34.1-42.9)
**Load (N)**	11.3 (10.1-12.2)	10.6 (10-11.3)	10.5 (9.5-12.8)	37.5 (36.2-41)
**Energy (N*mm)**	90.1 (44.7-109.9)	95.9 (74.1-111.2)	364.8 (299.3-435.1)	993.5 (882.8-1076.9)

	**Group VPWT**			
**Variable**	**Design 1 (n=5)**	**Design 2 (n=5)**	**Design 3 (n=5)**	**Design 4 (n=5)**
**Prior to yield**				
**Stiffness (mN/mm)**	34.5 (16-61)	21.7 (4.2-31.9)	3.1 (1.6-5.6)	13.6 (4.7-29.1)
**Yield**				
**Displacement (mm)**	0.7 (0.5-1)	1.2 (0.9-2.1)	4.5 (2.9-6.4)	3.7 (2.6-4.6)
**Load (N)**	2 (1.6-2.5)	2.2 (1.9-2.4)	2.4 (1.6-3)	7.4 (5.8-8.7)
**Energy (N*mm)**	1.06 (0.5-1.4)	1.8 (1.3-2.9)	7.9 (6.3-9.1)	18.26 (10.4-26.5)
**After Yield**				
**Stiffness (mN/mm)**	5 (1.8-8.8)	4.7 (3.8-5.7)	1.2 (0.9-1.6)	6.4 (5.6-7)
**Failure**				
**Displacement (mm)**	12.1 (9.8-14.4)	13.8 (12.3-15.5)	65.8 (47.9-69.8)	37.4 (35.6-38.8)
**Load (N)**	8.4 (7.6-9.8)	7.8 (7.6-8.2)	9.9 (8.2-11.3)	27.7 (24.6-29.5)
**Energy (N*mm)**	70.7 (56.7-89.2)	69.2 (57.6-84.2)	435.8 (237.1-573.8)	722.3 (605.2-785.1)
